# Emerging roles and mechanistic insights of ASRGL1 in tumorigenesis and cancer progression

**DOI:** 10.3389/fcell.2026.1955895

**Published:** 2026-10-21

**Authors:** Huiping Zeng, Zekang Li, Renhao Geng, Lujun Chen

**Affiliations:** 1 Changzhou Medical Center, Nanjing Medical University, Changzhou, Jiangsu, China; 2 Department of Tumor Biological Treatment, The Third Affiliated Hospital of Soochow University, Changzhou, Jiangsu, China; 3 Jiangsu Engineering Research Center for Tumor Immunotherapy, The Third Affiliated Hospital of Soochow University, Changzhou, Jiangsu, China; 4 Institute of Cell Therapy, Soochow University, Jiangsu, Changzhou, China; 5 The First People’s Hospital of Changzhou, Changzhou, Jiangsu, China

**Keywords:** asparagine metabolism, ASRGL1, metabolic reprogramming, tumor prognosis, tumorigenesis

## Abstract

Asparaginase and isoaspartyl peptidase 1 (ASRGL1), also known as ALP, CRASH, hASNase3, is a protein encoded by the ASRGL1 gene located at chromosome 11q12.3 in humans. It belongs to the N-terminal nucleophilic (Ntn) hydrolase superfamily and catalyzes the hydrolysis of L-asparagine and isoaspartyl dipeptides, thereby participating in amino acid metabolism and protein homeostasis regulation. In recent years, accumulating evidence has shown that ASRGL1 is aberrantly expressed in multiple human cancers, however, its biological and prognostic implications vary substantially among tumor types and are highly tumor-context dependent. This review systematically summarizes the structure and function of ASRGL1, its expression patterns and clinical relevance, the reported functional roles and proposed mechanisms in tumorigenesis and progression, and discusses its potential as a candidate biomarker for clinical prognosis prediction.

## 1 Introduction

Metabolic reprogramming is one of the hallmarks of malignant tumors. Tumor cells reconfigure metabolic pathways to meet the demands of rapid proliferation, including energy requirements and biosynthetic precursor supply (Mao et al., 2024; Lin et al., 2024; Hanahan and Weinberg, 2011). Dysregulation of amino acid metabolic homeostasis represents a core component of tumor metabolic reprogramming and exerts vital regulatory effects during tumor initiation and progression of malignant tumors (Ham et al., 2026; Ge et al., 2025). As an essential component of the amino acid metabolic network, asparagine metabolism plays a critical role in tumor cell growth and survival (Hanada et al., 2025; Yuan et al., 2024).

The activity of L-asparaginase has long been exploited therapeutically for the treatment of acute lymphoblastic leukemia. However, human asparaginase and isoaspartyl peptidase 1 (ASRGL1) itself has mainly been investigated as a potential alternative enzyme, and has not been established as a clinical therapeutic agent. Studies have also demonstrated its involvement in retinal degeneration and neurodegenerative diseases (Biswas et al., 2022; Zhou et al., 2021; Simula et al., 2026; Biswas et al., 2016; Xing et al., 2025). ASRGL1 exhibits enzymatic activity to hydrolyze L-asparagine and isoaspartyl dipeptides, catalyzing the conversion of L-asparagine into L-aspartate and ammonia (Wang et al., 2023; Xue et al., 2021). Moreover, it plays a complex and multifaceted role in the initiation and progression of solid tumors.

Recent advances in single-cell transcriptomics, metabolomics, and bioinformatics, together with analyses of large-scale public databases and clinical samples, have revealed the potential of ASRGL1 as a prognostic biomarker, and as a component of metabolism-related prognostic signatures, across multiple tumor types, including hepatocellular carcinoma, renal cell carcinoma, colorectal cancer and endometrial carcinoma (Xue et al., 2021; Li et al., 2023; Huang et al., 2024; Zhai et al., 2023).

This review summarizes the structure, functions, and emerging roles of ASRGL1 in tumor initiation and progression, focusing on its expression, prognostic value, molecular mechanisms, metabolic reprogramming, and tumor microenvironment crosstalk, to inform future basic research and clinical translation.

## 2 Molecular structure and biological characteristics of ASRGL1

ASRGL1 is a member of the N-terminal nucleophilic (Ntn) hydrolase superfamily (De Morais and De Souza, 2021). The gene is located on chromosome 11q12.3 and consists of seven exons and six introns, encoding a 308-amino-acid protein with a molecular weight of 31.9 kDa. Its structural core contains a conserved Ntn αββα fold. ASRGL1 requires a self-activation process to convert from an inactive precursor into an active enzyme (Bush et al., 2002; Li et al., 2016). Intramolecular processing between Gly167 and Thr168 exposes a new N-terminal Thr168 that serves as the catalytic nucleophile, generating the active enzyme with an αββα fold (Morais et al., 2020; Figure 1). ASRGL1 possesses both L-asparaginase and β-aspartyl peptidase activities, enabling it to catalyze the hydrolysis of L-asparagine and isoaspartyl dipeptides. L-asparagine is an important non-essential amino acid for the growth and development of both normal and tumor cells. The L-asparaginase activity of ASRGL1 not only converts L-asparagine into L-aspartate and ammonia, thereby influencing amino acid metabolism in tumor cells, but also selectively inhibits the phosphorylation of p70 S6 kinase (p70^s6k^) and eukaryotic initiation factor 4E-binding protein 1 (4E-BP1), ultimately suppressing tumor cell proliferation (Weidle et al., 2009). Isoaspartyl dipeptides are generated by non-enzymatic protein damage within cells, which can lead to protein misfolding, dysfunction, and reduced degradation. The β-aspartyl peptidase activity of ASRGL1 recognizes and cleaves isoaspartyl residues, thereby restoring protein function. Notably, it has been suggested that ASRGL1 may cooperate with isoaspartyl methyltransferase to alleviate the accumulation of potentially toxic isoaspartyl peptides in mammalian brain and other tissues (Garcia-Montojo et al., 2024). Through its involvement in amino acid recycling and the reduction of protein misfolding and aggregation, ASRGL1 helps maintain normal functions of the nervous and reproductive systems; its mutation or loss can lead to retinal degeneration (mouse model) and has been associated with infertility and neurodegenerative changes (Biswas et al., 2022; Fitzgerald et al., 2018; Liu et al., 2018). Although these studies have primarily focused on the nervous and reproductive systems, they provide important clues for understanding the role of ASRGL1 in regulating protein homeostasis and stress responses in tumor cells.

**FIGURE 1:**
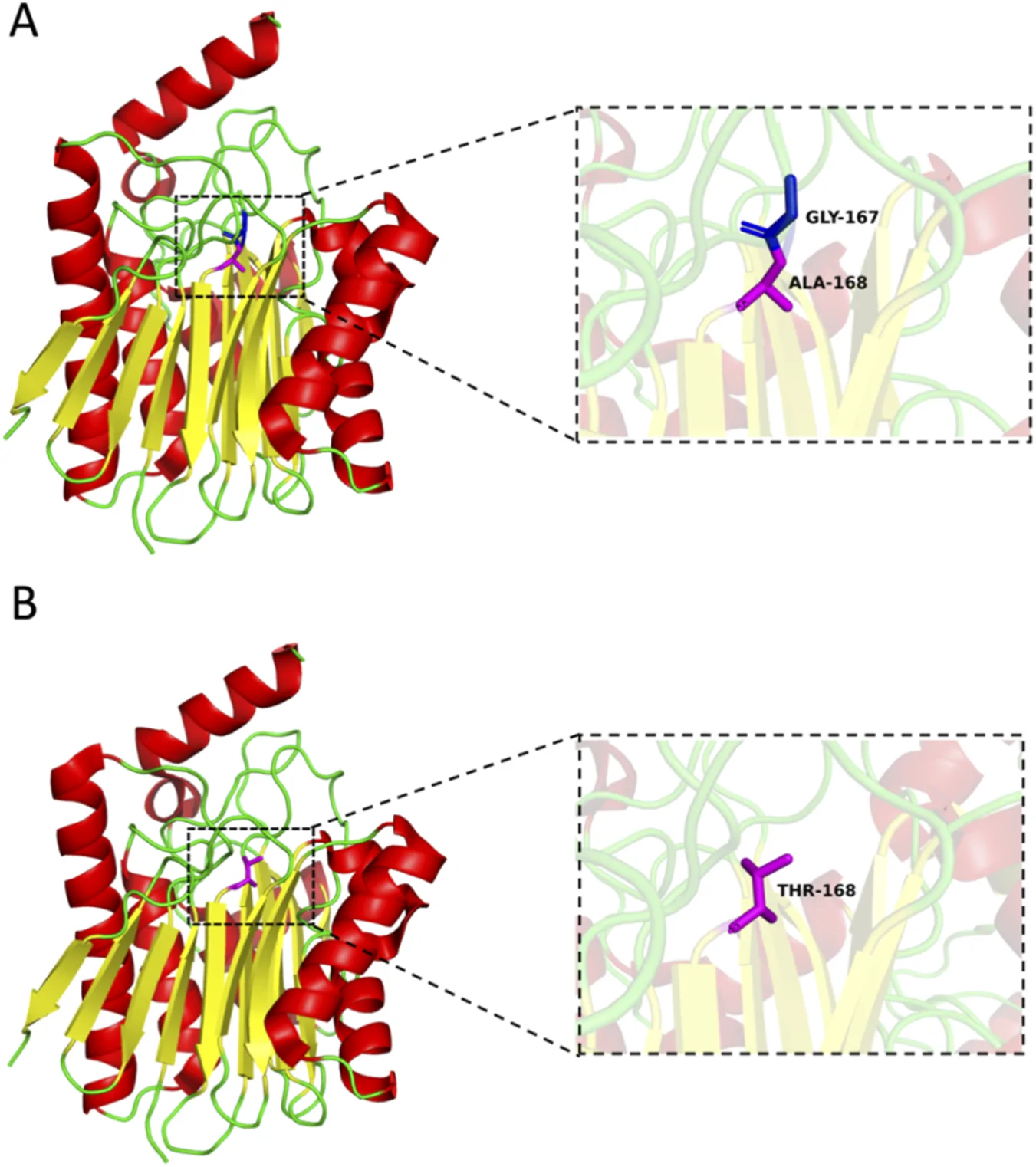
Schematic representation of ASRGL1 protein structure. **(A)** Inactive ASRGL1 (PDB ID: 3TKJ). Thr168 is mutated to Ala168, preventing self-cleavage. **(B)** Active ASRGL1 (PDB ID: 4ET0). Self-cleavage has occurred, and Thr168 becomes the new N-terminus. α subunit (red), β subunit (yellow), HGG loop (green).

## 3 Expression characteristics and clinical prognostic value of ASRGL1 in human cancers

ASRGL1 is predominantly expressed in the testis, brain, prostate, and proliferating endometrium (Weidle et al., 2009). Increasing evidence indicates that ASRGL1 is aberrantly expressed in various malignancies, including nasopharyngeal carcinoma, breast cancer, hepatocellular carcinoma, colorectal cancer, renal cell carcinoma, cervical cancer, and endometrial cancer (Feng et al., 2024; Lv et al., 2018; Eren Karanis et al., 2021). The expression profiles, functional tendencies, and clinical implications of ASRGL1 differ across tumor types, as summarized in Table 1.

**TABLE 1:** Expression patterns, functional roles, key mechanisms, and prognostic associations of ASRGL1 in human cancers.

Cancer type	Expression trend	Function	Key mechanisms	Prognostic association	References
Nasopharyngeal carcinoma	Mostly low expression (some studies report high)	Complex (low expression but knockdown suppresses tumors)	Low expression associated with EMT; knockdown inhibits proliferation, migration, induces G1/S arrest, apoptosis, ferroptosis	Low expression correlates with malignant phenotype	Su et al. (2024), Chen et al. (2019), Pei et al. (2025), Feng et al. (2024)
Breast cancer	Higher in ER/PR-positive,IDC	Pro-tumor (especially in metastasis)	Regulated by estrogen response element (ERE); promotes metastasis	No significant association with DFS or OS	Weidle et al. (2009), Evtimova et al. (2004), Eren Karanis et al. (2021), Loibl et al. (2021), Li et al. (2022)
Hepatocellular carcinoma	Higher than adjacent normal tissues	Pro-tumor	Cyclin B/CDK1 axis promotes G2/M progression; suppresses p53; associated with immune infiltration	High expression correlates with worse RFS, PFS, DSS, OS	Xue et al. (2021), Wang et al. (2023)
Colorectal cancer	Higher in highly metastatic cells	Functional evidence in selected cell models; discordant clinical association	High expression in low-risk group; enriched in lipid/vitamin metabolism pathways	Higher expression correlates with favorable prognosis	Weidle et al. (2009), Li et al. (2021), Chen et al. (2021)
Renal cell carcinoma	Lower in tumor tissues	Correlative prognostic association	Protective factor in a metabolism-related gene signature	Higher expression correlates with longer survival	Li et al. (2023)
Cervical cancer	Higher than adjacent normal tissues	Pro-tumor	Regulates CDK2/cyclin A2, Bax/Bcl-2; induces S-phase arrest and apoptosis	Proposed diagnostic biomarker for malignant progression of cervical lesions	Xu et al. (2019), Lv et al. (2018)
Prostate cancer	High transcriptional level	Pro-tumor (biochemical recurrence)	Possibly through amino acid metabolism or TMPRSS2-ERG fusion	High expression associated with biochemical recurrence and poor prognosis	Chu et al. (2018), Pudova et al. (2019)
Endometrial cancer	High in normal endometrium; heterogeneously low in cancer	Clinical/prognostic association	Involved in glutamine-asparagine and glucose metabolism; associated with p53-abnormal subtype	Low expression correlates with advanced stage, grade, lymph node metastasis, worse DSS	Lu and Broaddus, (2020), Crosbie et al. (2022), Edqvist et al. (2015), Fonnes et al. (2018), Huvila et al. (2018)

### 3.1 Nasopharyngeal carcinoma

Nasopharyngeal carcinoma (NPC) is a malignancy with distinct geographical distribution, being highly prevalent in Southeast Asia and Southern China. Despite advances in radiotherapy and chemotherapy, locoregional recurrence and distant metastasis remain major causes of treatment failure (Su et al., 2024; Chen et al., 2019). In NPC, the expression levels and functional roles of ASRGL1 remain controversial, with different studies reporting seemingly contradictory findings. Feng *et al.* detected high ASRGL1 expression in NPC tissues using RT-qPCR and western blotting (Feng et al., 2024). In contrast, Pei *et al.* based on single-cell transcriptomic and metabolomic analyses, reported that ASRGL1 is primarily expressed in fibroblasts, epithelial cells, and myeloid cells, where its expression levels are significantly lower than those in adjacent normal tissues, and that low expression is closely associated with the malignant phenotype of NPC (Pei et al., 2025). Despite these conflicting expression observations, preliminary functional studies imply that ASRGL1 may be linked to tumor-cell growth and cell-death phenotypes in NPC. (Pei et al., 2025; Feng et al., 2024). This suggests that the mechanism of ASRGL1 action is tissue-specific and context-dependent. Its aberrant expression is closely linked to the regulation of NPC cell proliferation, invasion, and cell death pathways, positioning it as a potential diagnostic biomarker or therapeutic target, although further studies are urgently needed to elucidate its complex mechanisms.

### 3.2 Breast cancer

Breast cancer is the most commonly diagnosed malignancy among women worldwide. Hormone receptor status (ER/PR) is a key determinant of prognosis and treatment strategy, with ER/PR-positive tumors generally exhibiting better clinical outcomes than triple-negative or hormone-negative subtypes (Loibl et al., 2021; Li et al., 2022). In breast cancer, ASRGL1 expression is regulated by sex hormones and is closely associated with hormone receptor status. Notably, an estrogen response element (ERE) has been identified in the promoter region of the ASRGL1 gene, through which sex hormones induce ASRGL1 expression in BT474 breast cancer cells (Evtimova et al., 2004). Compared with normal mammary epithelial cells and primary breast cancer cells, ASRGL1 mRNA is highly expressed in metastatic breast cancer cells (Evtimova et al., 2004; Weidle et al., 2009). Eren Karanis *et al.* performed immunohistochemistry on 148 invasive ductal carcinoma (IDC) samples and 105 non-neoplastic breast tissue specimens, and found that ASRGL1 expression levels were higher in estrogen receptor (ER)/progesterone receptor (PR)-positive IDC than in normal breast tissue, whereas ASRGL1 expression was downregulated in ER/PR-negative IDC, suggesting a positive correlation between ASRGL1 expression and ER/PR status. It is well established that patients with ER/PR-positive IDC have a better prognosis. Although loss of ASRGL1 in ER/PR-negative tumors may indicate poor prognosis, further analyses revealed that ASRGL1 expression levels were not significantly associated with clinicopathological features such as tumor size, grade, stage, lymph node status, or distant metastasis, nor were they correlated with disease-free survival or overall survival (Eren Karanis et al., 2021). Collectively, these findings suggest that while ASRGL1 interacts with hormone receptor pathways and may play an important role in breast cancer progression, particularly in metastasis, its reliability as a standalone prognostic marker in breast cancer is limited. Thus, combining ASRGL1 with other molecular markers may be necessary for comprehensive prognostic assessment. Future studies could further explore its potential value in molecularly targeted therapy for breast cancer.

### 3.3 Hepatocellular carcinoma

Hepatocellular carcinoma (HCC) is the third leading cause of cancer-related mortality worldwide, with rising incidence in many regions. Despite the availability of multikinase inhibitors and immune checkpoint inhibitors, the prognosis for advanced-stage HCC patients remains poor, underscoring the urgent need for novel biomarkers and therapeutic targets (Vogel et al., 2022; Llovet et al., 2021). ASRGL1 expression levels are significantly higher in HCC tissues than those in adjacent normal tissues, and its upregulation positively correlates with tumor pathological grade, T stage, clinical stage, and lymph node metastasis (Xue et al., 2021; Wang et al., 2023). Kaplan–Meier survival analysis showed that high ASRGL1 expression was associated with poorer recurrence-free survival (RFS), progression-free survival (PFS), disease-specific survival (DSS), and overall survival (OS). In addition, Zhong *et al.* constructed a ceRNA network and found that ASRGL1 is one of the 29 genes associated with survival and prognosis in HCC, and it is closely correlated with the infiltration levels of multiple immune cell types (Zhong et al., 2023). Furthermore, co-expression network analysis based on the LinkedOmics database and gene set enrichment analysis (GSEA) indicate that ASRGL1-associated genes are mainly enriched in pathways related to DNA replication, microtubule cytoskeleton organization, spliceosome development, and homologous recombination (Xue et al., 2021; Wang et al., 2023).

Collectively, these findings indicate that elevated ASRGL1 expression in HCC is closely associated with adverse clinicopathological features and poor survival, supporting its potential as a prognostic biomarker.

### 3.4 Colorectal cancer

Colorectal cancer (CRC) is the second leading cause of cancer death globally. Metastasis is the primary cause of mortality in CRC patients, and identification of metastasis-associated molecular markers is critical for risk stratification and personalized therapy (Biller and Schrag, 2021; Eng et al., 2024). In CRC, ASRGL1 expression is closely associated with tumor metastatic potential and patient prognosis. Functional studies have revealed that ASRGL1 mRNA expression levels are significantly higher in highly metastatic KM12L4A cells than in moderately metastatic KM12SM cells. Knockdown of ASRGL1 markedly suppressed the proliferation of KM12L4A cells but did not significantly affect KM12SM cells, suggesting that invasive cancer cells with high ASRGL1 expression possess a selective growth advantage (Weidle et al., 2009). Bioinformatics analyses based on public databases have further revealed the prognostic value of ASRGL1. Li *et al.* identified Th17 cell-related hub genes (including KRT23, ULBP2, ASRGL1, SERPINA1, SCIN, and SLC28A2) associated with prognosis in colorectal cancer from the TCGA database, and found that patients with high ASRGL1 expression in the low-risk group had a better prognosis (Li et al., 2021; Chen et al., 2021). Among these, ASRGL1 was highly expressed in the low-risk group and correlated with enrichment of lipid and vitamin metabolism pathways as well as favorable prognosis.

Collectively, these apparently discordant experimental and clinical observations indicate that the functional role of ASRGL1 in CRC remains unresolved. Although ASRGL1 knockdown suppresses proliferation of highly metastatic CRC cells *in vitro*, higher ASRGL1 expression is associated with favorable prognosis in patient datasets, further studies are required to clarify the relationship between its expression levels, cellular functions and prognostic associations.

### 3.5 Renal cell carcinoma

Renal cell carcinoma (RCC) is characterized by profound metabolic reprogramming, with alterations in glucose, lipid, and amino acid metabolism serving as hallmarks of disease progression. The growing recognition of metabolic vulnerabilities has opened new avenues for therapeutic intervention (Hsieh et al., 2017; Linehan et al., 2019). In RCC, metabolic abnormalities are a hallmark of tumor development and progression. Li *et al.* constructed a prognostic model based on six metabolism-related genes (ACAA2, ACAT1, ASRGL1, AKR1B10, ABCC2, and ANGPTL4) using the TCGA database, and found that ASRGL1 expression was significantly downregulated in RCC subsets with a low-metabolism phenotype and poor prognosis (Li et al., 2023). Further analysis revealed that ASRGL1 was highly expressed in low-risk patients with favorable prognosis, and that high ASRGL1 expression correlated with longer patient survival. Immunohistochemical analysis further confirmed that ASRGL1 exhibited strong or moderate staining intensity in normal kidney tissues, whereas staining intensity was markedly reduced in tumor tissues, consistent with the association between low ASRGL1 expression and poorer survival. Collectively, these findings indicate that ASRGL1, as a component of a metabolism-related prognostic signature in RCC, contributes to risk stratification and prognosis prediction, and may offer auxiliary diagnostic value for distinguishing RCC from normal tissues based on expression differences.

### 3.6 Cervical cancer

Cervical cancer remains a major cause of cancer-related morbidity among women worldwide, particularly in regions with limited screening coverage. Although HPV vaccination and early detection have reduced its incidence in many countries, advanced or recurrent cervical cancer still carries a poor prognosis and lacks effective targeted therapies, necessitating the discovery of new molecular targets (Cohen et al., 2019; Siegel et al., 2025). In cervical cancer, ASRGL1 expression levels are significantly higher than those in adjacent normal tissues (Lv et al., 2018). Furthermore, Xu *et al.* through RNA-seq screening and RT-qPCR validation, identified ASRGL1 as a potential biomarker for indicating malignant progression stage of cervical lesions, exhibiting high diagnostic sensitivity and specificity (Xu et al., 2019). Collectively, these lines of evidence indicate that ASRGL1 is closely associated with the development and progression of cervical cancer. Molecular targeted therapy directed against ASRGL1 may offer novel clinical strategies, and this gene holds promise as a potential target for cervical cancer diagnosis and gene therapy (Xu et al., 2019; Lv et al., 2018).

### 3.7 Prostate cancer

Prostate cancer is the most frequently diagnosed non-skin malignancy in men. While localized disease is often curable by surgery or radiotherapy, a subset of patients experience biochemical recurrence after radical prostatectomy, which complicates clinical decision-making regarding adjuvant therapy. Prognostic gene signatures that can reliably predict recurrence risk are therefore urgently needed to guide personalized treatment (Sandhu et al., 2021; Rebello et al., 2021). Chu *et al.* developed a risk-scoring model based on eight genes (CHST1, ACOX1, CTBS, GNPNAT1, NAGLU, LPIN3, ASRGL1, and HMGCS2) using the TCGA database. This model demonstrated significantly better predictive performance for biochemical recurrence in prostate cancer than any single clinical parameter (Chu et al., 2018). Among these genes, ASRGL1 was identified as a key predictor of biochemical recurrence: its high expression was significantly associated with poor prognosis and positively correlated with TNM stage, Gleason score, and lymph node invasion, while remaining independent of patient age. Although the precise molecular mechanisms of ASRGL1 in prostate cancer remain unclear, current evidence suggests that its high transcriptional levels may promote tumor cell proliferation and recurrence by influencing amino acid metabolism or through interaction with the TMPRSS2-ERG fusion gene (Pudova et al., 2019). The TMPRSS2-ERG fusion subtype is the most common molecular subtype of prostate cancer. Pudova *et al.* based on TCGA data analysis of locally advanced lymph node-negative prostate cancer of this subtype, also identified ASRGL1 as playing an important role in prognosis, suggesting that ASRGL1 may serve as a potential target for precision prognosis and therapy of this subtype in prostate cancer are closely associated with prognosis (Pudova et al., 2019). Nevertheless, its upstream and downstream regulatory networks remain to be thoroughly explored.

### 3.8 Endometrial cancer

Endometrial cancer (EC) is the most common gynecological malignancy in developed countries. Its incidence has been rising in parallel with increasing rates of obesity and metabolic disorders. Although most patients present with early-stage disease and have a favorable prognosis, accurate preoperative assessment of lymph node metastasis remains challenging, and reliable biomarkers are needed to guide surgical staging and adjuvant therapy (Lu and Broaddus, 2020; Crosbie et al., 2022). In EC, ASRGL1 is highly expressed in normal endometrial tissue but exhibits heterogeneous loss in EC tissues. Multiple studies consistently demonstrate that low ASRGL1 expression is closely associated with adverse clinicopathological parameters, including higher pathological stage, grade, lymph node status, and depth of myometrial invasion, and independently predicts lymph node metastasis and worse disease-specific survival. Edqvist *et al.* in a two-cohort study confirmed that ASRGL1 is highly expressed in normal endometrium but heterogeneously lost in endometrioid endometrial carcinoma (EEC) (Edqvist et al., 2015). Patients with low ASRGL1 expression had significantly shorter DSS than those with high expression, and both univariate and multivariate analyses suggested that ASRGL1 may serve as a potential independent prognostic factor for DSS in EEC. Fonnes *et al.* further demonstrated in a prospective cohort study that ASRGL1 is highly expressed in premalignant lesions but is frequently low or absent in metastatic lesions (Fonnes et al., 2018). Loss or low expression was more common in high-grade than in low-grade tumors, and ASRGL1 showed differential expression across pathological stage, grade, histological type, lymph node status, depth of myometrial invasion, and estrogen receptor status. Notably, the concordance rate of ASRGL1 expression between preoperative curettage and postoperative surgical specimens reached 85%, providing a reliable basis for preoperative risk assessment.

In terms of clinical prognostic model development, Huvila *et al.* performed immunohistochemistry on 306 EEC tissues using multiple established markers (ER, PR, HER2, Ki-67, MLH1, p53) and two novel markers (L1CAM, ASRGL1) (Huvila et al., 2018). LASSO regression analysis identified that combined ASRGL1 and p53 immunohistochemistry served as an independent predictor of survival in endometrioid endometrial carcinoma. Based on the finding that low ASRGL1 expression independently predicts lymph node metastasis, Huang *et al.* constructed a five-gene prediction model (including ASRGL1, RHEX, SCGB2A1, SOX17, and STX18) for risk assessment of lymph node metastasis in early-stage endometrioid adenocarcinoma (Huang et al., 2019; Fonnes et al., 2018).

Of note, although ASRGL1 has demonstrated prognostic value across multiple studies, its independent prognostic efficacy may be influenced by the tumor’s molecular background. Loukovaara *et al.* performed a retrospective analysis of 775 endometrial cancer patients and found that molecular classification was the only independent prognostic factor (Loukovaara et al., 2024). Although low ASRGL1 expression was significantly associated with the p53-abnormal subtype, its independent prognostic value was attenuated after multivariable adjustment. This finding suggests that when evaluating the clinical significance of ASRGL1, a comprehensive judgment integrating the molecular subtype of the tumor is warranted.

## 4 Molecular regulatory mechanism of ASRGL1

ASRGL1 can drive tumor progression through multiple pathways, including regulation of cell cycle progression, apoptosis-related signaling pathways, and metabolic reprogramming (Figure 2).

**FIGURE 2:**
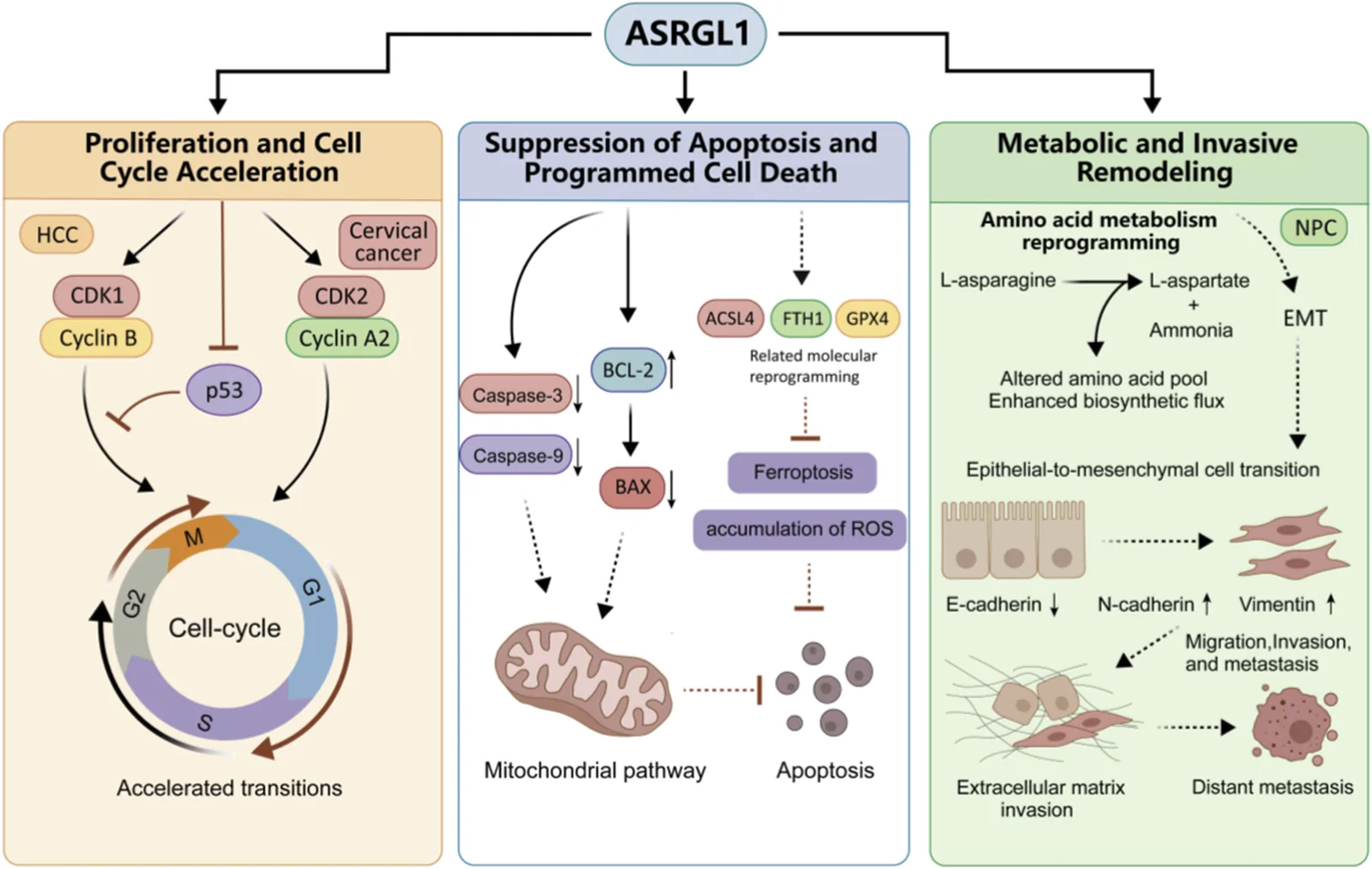
Reported functional effects and proposed mechanisms of ASRGL1 in cancer. Solid arrows indicate experimentally supported functional relationships; dashed arrows indicate correlative associations or proposed mechanisms. Experimentally supported effects include promotion of cell-cycle progression (G2/M arrest in HCC, S-phase arrest in cervical cancer, and G1/S arrest in NPC after ASRGL1 knockdown), suppression of apoptosis via the mitochondrial pathway (Bax/Bcl-2 balance and cleaved caspase-3/9), and enhancement of proliferation, migration, and invasion. Correlative or proposed relationships include amino acid metabolism reprogramming (L-asparagine/L-aspartate axis), EMT-related marker changes, ferroptosis-related molecular changes (ACSL4, FTH1, GPX4, ROS accumulation).

### 4.1 Cell cycle regulation

Dysregulation of the cell cycle is one of the key hallmarks of cancer development (Knudsen et al., 2025; Wan et al., 2026; Chen et al., 2026). ASRGL1’s role in cell cycle regulation was first elucidated in HCC. Wang *et al.* demonstrated that ASRGL1 knockdown caused HCC cells to arrest in the G2/M phase. Mechanistically, ASRGL1 knockdown promoted p53 protein expression, reduced the levels of Cyclin B and Cyclin-dependent kinase 1(CDK1) proteins, and disrupted the interaction between Cyclin B and CDK1 (Wang et al., 2023). Importantly, overexpression of CDK1 reversed the ASRGL1 knockdown–induced decreases in HepG2 cell proliferation, migration, and invasion, confirming that ASRGL1 promotes HCC tumorigenesis in a CDK1-dependent manner. In NPC, Feng *et al.* silenced ASRGL1 via lentiviral shRNA vectors and observed suppressed proliferative activity in C666-1 and SUN-1 cell lines. Cell cycle analysis revealed elevated proportions of cells in the G1 and G2 phases alongside a reduced S-phase cell population (Feng et al., 2024). These findings indicate that ASRGL1 ablation disrupts of NPC cells proliferation, predominantly via inducing the G1 checkpoints arrest. In the cervical cancer SiHa cell line, Lv *et al.* reported that ASRGL1 knockdown caused cell-cycle arrest in the S phase, along with decreased expression of Cyclin-dependent kinase 2(CDK2) and Cyclin A2 proteins. Meanwhile, Bcl-2 associated X protein (Bax) expression was increased, whereas B cell lymphoma 2(Bcl-2) expression was decreased (Lv et al., 2018). Bioinformatic analyses further validated the close correlation between ASRGL1 and cell cycle regulation. Zhong *et al.* constructed a circRNA-miRNA-mRNA regulatory network in HCC, and identified that ASRGL1, together with WDR76, E2F3 and POGK, predominantly activates pathways governing cell cycle progression and the DNA damage response (Zhong et al., 2023). These findings suggest that ASRGL1 may promote proliferation in different tumors by regulating distinct cell-cycle checkpoints.

### 4.2 Regulation of apoptosis

The anti-apoptotic function of ASRGL1 has been proposed as a mechanism that facilitates tumor cell survival. In cervical cancer SiHa cells, ASRGL1 knockdown upregulated Bax expression while downregulating Bcl-2, and was accompanied by reduced levels of CDK2 and cyclin A2. This indicates that ASRGL1 suppresses the mitochondria-mediated apoptotic pathway by modulating the balance of Bcl-2 family proteins (Lv et al., 2018). Mechanistic research conducted by Feng *et al.* has yielded more in-depth findings in NPC. Treatment with shASRGL1 elevated the proportions of cells undergoing early and late apoptosis, and increased the expression of cleaved caspase-3 and cleaved caspase-9, demonstrating that ASRGL1 exerted its biological effects via the intrinsic (mitochondrial) apoptotic pathway (Feng et al., 2024). Furthermore, this study demonstrated for the first time that ASRGL1 knockdown triggered reactive oxygen species accumulation and ferroptosis, accompanied by altered expression of ACSL4 and FTH1 as well as decreased glutathione peroxidase 4 (GPX4) levels. These results suggested that ASRGL1 may participate in the crosstalk regulation of multiple cell death modalities through modulating oxidative stress and lipid peroxidation.

### 4.3 Regulation of cell proliferation, invasion and migration

The effects of ASRGL1 on the proliferative, apoptotic, invasive and migratory capacities of tumor cells have been validated across multiple cancer types. In HCC, ASRGL1 knockdown suppressed the proliferation, migration and invasion of HepG2 and Li-7 cells while concomitantly inducing cellular apoptosis; *in vivo* experiments further confirmed that ASRGL1 silencing markedly impeded tumor growth (Wang et al., 2023). Conversely, ASRGL1 overexpression promoted the proliferative, migratory and invasive phenotypes of HepG2 cells. In NPC, shRNA-mediated ASRGL1 interference significantly impaired the viability, migration and invasion of C666-1 and SUN-1 cells (Feng et al., 2024). Consistent with these findings, silencing ASRGL1 also led to a prominent decline in the proliferative potential of cervical cancer SiHa cells (Lv et al., 2018). Pei *et al.* conducted integrative analyses of single-cell transcriptomics and metabolomics and found that genes highly expressed in ASRGL1-positive cells were significantly enriched in the epithelial-mesenchymal transition (EMT) pathway (Pei et al., 2025). Upon ASRGL1 knockdown in NPC cells, detection of altered expression levels of E-cadherin and N-cadherin verified that silencing ASRGL1 suppress the EMT program. These observations suggest that ASRGL1 may be involved in EMT regulation in NPC cells and may contribute to tumor cell migration and invasion; however, the causal relationship between ASRGL1 and EMT has not been fully established.

### 4.4 ASRGL1 in metabolic reprogramming

The association between ASRGL1 and metabolic reprogramming has been confirmed in multiple studies. In NPC cells, studies have shown that knockdown of ASRGL1 (shASRGL1) reduces ATP levels, suggesting that ASRGL1 may be involved in the regulation of energy metabolism in tumor cells (Feng et al., 2024). In addition, in a metabolic subtyping study of RCC, ASRGL1 was downregulated in the low-metabolism subgroup, which also exhibited poorer prognosis and greater invasiveness (Li et al., 2023).

As a member of the asparaginase family, ASRGL1 exerts its core biological function by regulating amino acid metabolism. Using amino acid metabolomic profiling, Pei *et al.* demonstrated that knockdown of ASRGL1 markedly attenuated the utilization of multiple amino acids NPC cells (Pei et al., 2025). Subsequent GC-MS-based metabolomic analyses further detected substantial metabolic dysregulation upon ASRGL1 silencing, suggesting that ASRGL1 may contribute to maintaining the amino-acid metabolic homeostasis of tumor cells.

This observation aligns well with the enzymatic property of ASRGL1: the enzyme catalyzes the hydrolysis of L-asparagine to supply abundant amino acid substrates, thereby meeting the high metabolic demands for rapid neoplastic proliferation. In lung cancer cells, Valdez-Arellanes *et al.* observed that treatment with Coleus hadiensis extract suppressed ASRGL1 expression, which was accompanied by reprogramming of amino acid catabolism (Valdez-Arellanes et al., 2026). In another study, Zhai *et al.* incorporated ASRGL1 into a glutamine metabolism-related prognostic signature, further supporting an association between ASRGL1 and glutamine/amino acid metabolic networks (Zhai et al., 2023). In endometrial cancer, Zhai et al. were the first to incorporate ASRGL1 into the glutamine metabolism network, and found that low ASRGL1 expression cooperated with high PHGDH expression, co-enriching the glutamine-to-asparagine metabolic pathway and DNA repair abnormalities (Zhai et al., 2023). *In vitro* experiments confirmed that a PHGDH inhibitor significantly suppressed the proliferation and migration of cells with low ASRGL1 expression, suggesting that ASRGL1 may participate in tumor energy homeostasis remodeling through the regulation of amino acid metabolism. Focusing on glucose metabolism, Jiang et al. identified six prognostic genes (ASRGL1, SLC38A3, SLC2A1, ALDH1B1, GAD1, and GLYATL1) by univariate Cox regression and LASSO regression (Jiang et al., 2025). ASRGL1 was highly expressed in ciliated and epithelial cells and negatively correlated with key glycolytic genes such as SLC2A1. Moreover, overexpression of SLC38A3 promoted glucose uptake and lactate production in tumor cells, further suggesting a link between ASRGL1 and tumor metabolic reprogramming. Huang *et al.* performed subpopulation analysis of M2 macrophages and identified ASRGL1 as a pivotal gene linking immunity and tumor metabolism. In colorectal cancer, M2 macrophages exhibit metabolic heterogeneity and can be divided into two subsets; among them, Cluster 2 with high metabolic activity correlates with poorer prognosis. ASRGL1, one of eight metabolic genes, was utilized to establish a prognostic prediction model (Huang et al., 2024). Via single-cell RNA sequencing analysis, Desterke *et al.* discovered that ASRGL1 is upregulated in colonic tissues from patients with ulcerative colitis. As one of 22 metabolic enzymes, ASRGL1 participated in the transcriptional activation of metabolic programs, further indicating its potential function in inflammation-associated metabolic reprogramming (Desterke et al., 2024).

Importantly, although the catalytic properties of ASRGL1 are biochemically well established, current cancer studies have not conclusively demonstrated that its catalytic activity directly drives the observed malignant phenotypes. Changes in ATP levels, amino acid utilization, or metabolomic profiles following ASRGL1 depletion establish an association with metabolic alterations but do not demonstrate catalytic causality. Catalytic-mutant and metabolite-rescue experiments will be required to test whether ASRGL1 enzymatic activity is causally linked to cancer metabolic reprogramming.

### 4.5 Association with the immune microenvironment

At the tumor microenvironment level, using the TIMER database, Xue *et al.* demonstrated that ASRGL1 expression in HCC was significantly correlated with the infiltration levels of B cells, CD4^+^T cells, macrophages, neutrophils and dendritic cells (Xue et al., 2021). Further analyses revealed that high ASRGL1 expression conferred a 1.56-fold increased risk of OS deterioration in HCC patients, indicating that ASRGL1 expression is associated with immune-cell infiltration and correlates with prognosis in HCC. ASRGL1 expression was also reported to correlate with multiple immune cell populations in HCC (Zhong et al., 2023). These findings are primarily correlative, and direct experimental evidence for ASRGL1-mediated immune regulation remains lacking. Collectively, the reported biological associations and clinical implications of ASRGL1 across human cancers are schematically summarized in Figure 3, illustrating its context-dependent roles in tumor cell biology and its current status as a candidate prognostic biomarker.

**FIGURE 3:**
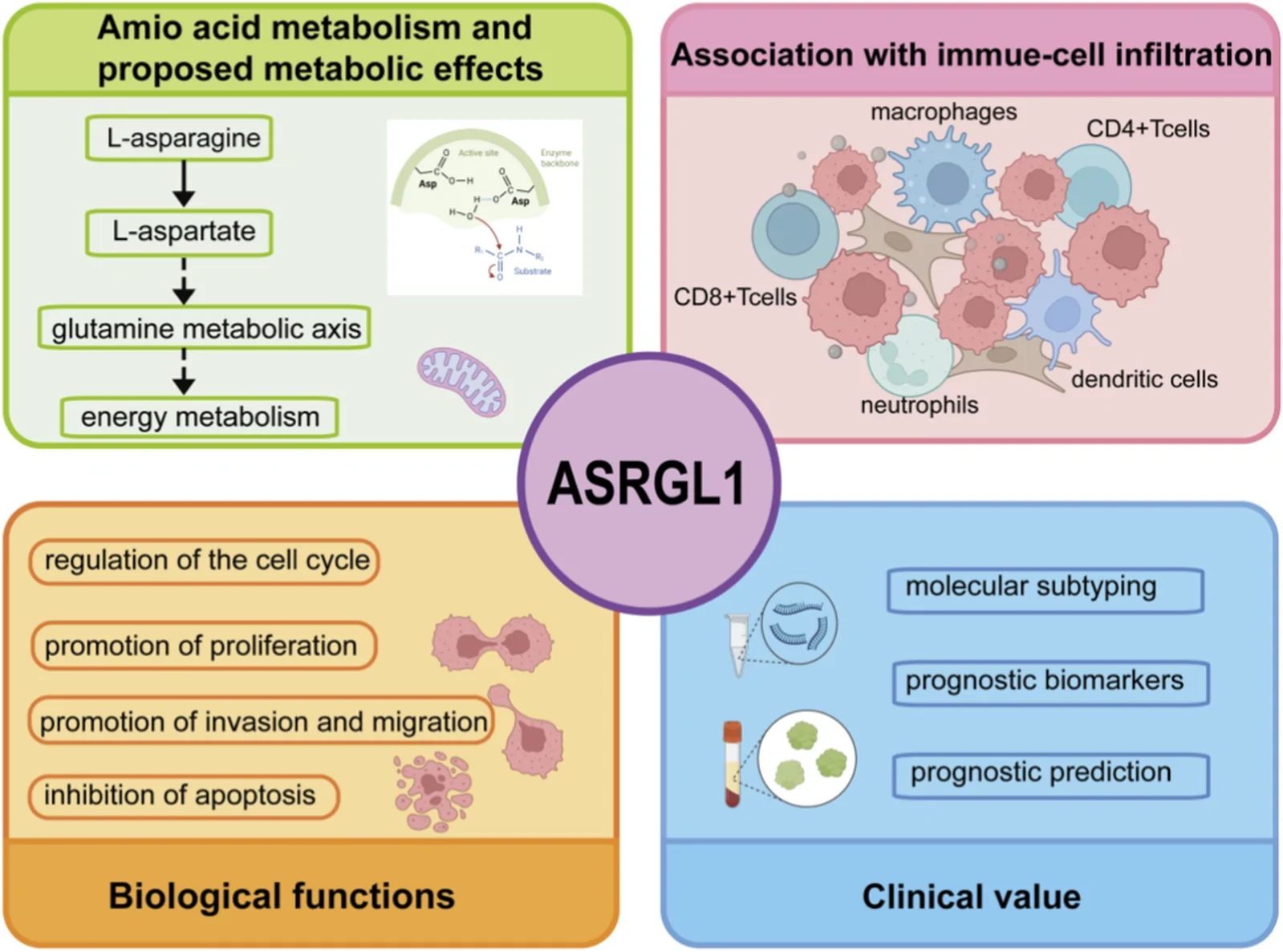
Summary of reported biological associations and clinical value of ASRGL1. ASRGL1 expression has been associated with metabolic features, immune-cell infiltration, cell-cycle progression, proliferation, invasion, migration, and apoptosis in a context-dependent manner. Clinically, ASRGL1 has been incorporated into prognostic and molecular-subtyping models, but its independent clinical utility requires prospective validation.

## 5 Discussion

ASRGL1 is an N-terminal nucleophile hydrolase that catalyzes the hydrolysis of L-asparagine and isoaspartyl dipeptides, and its enzymatic properties have been well characterized at the biochemical level. However, the central issue that emerges from the current literature is that the biological significance of ASRGL1 in cancer cannot be understood through its enzymatic activity alone. Across different tumor types, ASRGL1 exhibits markedly heterogeneous expression patterns and apparently contradictory functional associations. In HCC, ASRGL1 is upregulated and its expression correlates with adverse clinical outcomes; knockdown suppresses proliferation, migration, and invasion, and CDK1 overexpression partially reverses these effects. In NPC and cervical cancer, ASRGL1 depletion inhibits proliferation and induces cell-cycle arrest and apoptosis in selected cell models, yet NPC expression data remain discordant between studies. In CRC, ASRGL1 supports growth in highly metastatic KM12L4A cells, whereas higher ASRGL1 expression has been associated with favorable prognosis in bioinformatic models. In endometrial cancer and RCC, low ASRGL1 expression is associated with poor prognosis, but this association does not establish a tumor-suppressive function. These findings collectively indicate that ASRGL1 should not be regarded as a uniform oncogenic driver. Rather, its biological and prognostic implications appear highly context dependent, and the apparent contradictions across cancer types likely reflect differences in tissue origin, molecular subtype, differentiation state, metabolic context, and the distinction between tumor-cell-intrinsic functional experiments and bulk-tissue prognostic associations.

At the metabolic level, the relationship between ASRGL1 enzymatic activity and cancer-associated metabolic reprogramming remains unresolved. ASRGL1 depletion has been associated with reduced ATP levels, altered amino acid utilization, and widespread metabolomic changes in selected models. These observations are consistent with the known catalytic function of ASRGL1, but they do not demonstrate that its enzymatic activity directly drives malignant phenotypes. Although Thr168 has been established as the catalytic N-terminal nucleophile of ASRGL1, whether catalytic inactivation of ASRGL1 abolishes its cancer-associated phenotypes has not yet been directly demonstrated. Catalytic-mutant and metabolite-rescue experiments are required to establish whether ASRGL1 enzymatic activity is causally linked to tumor metabolic reprogramming, and until such evidence is available, the metabolic role of ASRGL1 in cancer should be considered a proposed model rather than an established mechanism.

The functional evidence for ASRGL1 in cancer varies considerably in strength across different biological processes. The CDK1 rescue experiment in HCC provides relatively strong support for a CDK1-dependent functional axis, whereas EMT-related findings in NPC are based mainly on expression changes of E-cadherin and N-cadherin after ASRGL1 knockdown and should be regarded as proposed mechanisms. The anti-apoptotic role of ASRGL1 has been suggested by changes in Bax, Bcl-2, cleaved caspase-3, cleaved caspase-9, and GPX4, but direct downstream targets remain to be defined. Similarly, the association between ASRGL1 expression and immune-cell infiltration has been reported in multiple bioinformatic analyses, but current evidence is primarily correlative, and direct experimental demonstration of ASRGL1-mediated immune regulation is lacking. Whether ASRGL1 modulates immune-cell recruitment or function, or predicts responses to immune checkpoint inhibitors, remains to be determined.

ASRGL1 has also been incorporated into multiple metabolism-related prognostic models and has shown associations with survival, molecular subtype, and immune-cell infiltration. In HCC, elevated ASRGL1 expression is associated with poor outcomes. In RCC, ASRGL1 was identified as a protective factor in a six-gene prognostic model. In endometrial cancer, however, the independent prognostic value of ASRGL1 was attenuated after adjustment for molecular classification, indicating that its clinical significance should be evaluated in the context of molecular subtype. These findings support ASRGL1 as a candidate biomarker, but most evidence is derived from retrospective bioinformatic analyses and bulk-tissue transcriptomic data. Prospective validation and integration with established molecular classifiers are required before ASRGL1 can be considered a clinically useful prognostic or predictive marker.

Several key questions remain to be addressed. First, the upstream regulatory mechanisms that govern ASRGL1 expression in a tissue-specific manner are largely unknown. How transcription factors, non-coding RNAs, and epigenetic modifications contribute to the divergent expression patterns of ASRGL1 across cancer types requires systematic investigation. Second, the relationship between ASRGL1 enzymatic activity and its cancer-associated phenotypes remains unresolved; catalytic-mutant and metabolite-rescue experiments will be essential. Third, the role of ASRGL1 in the tumor immune microenvironment requires direct experimental investigation, including its potential to modulate immune-cell recruitment and function and its predictive value for immunotherapy responses. Fourth, therapeutic targeting of ASRGL1 remains in its infancy; small-molecule inhibitors or specific antibodies targeting ASRGL1 have not yet been developed or validated in preclinical models. Finally, the translational potential of ASRGL1 for early diagnosis and liquid biopsy warrants further exploration, but large-scale prospective cohort studies are needed to validate the prognostic models in which ASRGL1 is included. Addressing these questions will require integrating biochemical, functional, and clinical approaches, and will be essential for determining whether ASRGL1 represents a therapeutically actionable vulnerability or primarily a context-dependent biomarker.

## 6 Conclusion

Current evidence supports ASRGL1 as an emerging, context-dependent cancer-associated metabolic enzyme and candidate biomarker. Its enzymatic properties are biochemically well established, and functional studies have supported context-dependent roles in cell-cycle progression, apoptosis, proliferation, invasion, EMT, and metabolic adaptation. However, many clinical associations remain correlative, and the causal contribution of ASRGL1 enzymatic activity to malignant phenotypes has not been conclusively demonstrated. Whether ASRGL1 represents a therapeutically actionable vulnerability remains to be established through rigorous mechanistic and preclinical studies. Future work should prioritize catalytic-mutant and metabolite-rescue experiments, context-specific functional models, and prospective clinical validation.
